# Supplementary material for: Direct programming of confined surface phonon polariton resonators with the plasmonic phase-change material In3SbTe2
Source: Nat Commun. 2024 Apr 24;15:3472. doi: 10.1038/s41467-024-47841-0 (PMC11043413; doi:10.1038/s41467-024-47841-0)
Supplement: Supplementary file 1 — Supplementary Information [file 41467_2024_47841_MOESM1_ESM.pdf]

Title:

Direct programming of confined Surface Phonon Polariton Resonators with the plasmonic Phase-Change Material In_3SbTe_2

*Author(s), and Corresponding Author(s)**

Lukas Conrads[+,], Luis Schüler[+], Konstantin G. Wirth, Matthias Wuttig, Thomas Taubner[#]*

+ authors contributed equally

This PDF file includes:

Supplementary Note 1: Infrared optical properties of In_3SbTe_2 (IST)

Supplementary Note 2: Polariton fitting

Supplementary Note 3: SPhP Dispersion

Supplementary Note 4: Writing procedure of confined resonators

Supplementary Note 5: Images for different diameters

Supplementary Note 6: Field simulations of the cavities with varied diameters

Supplementary Note 7: Quality factor of the resonators

Supplementary Note 8: Analytical model of circular resonators

Supplementary Note 9: Resonator arrays investigated in the far-field

Supplementary Note 10: Comparative Study with previous literature

Supplementary Note 11: Reamorphization of cavities

Supplementary Note 12: Hyperbolic Phonon Polariton cavities with hBN

References

Supplementary Note 1: Infrared optical properties of In_3SbTe_2 (IST)

The dielectric function of IST is obtained with infrared spectroscopy and ellipsometry. The details are taken from Heßler et al.[1]:

In general, we apply the 'Tauc-Lorentz Dispersion formula' by assuming a Lorentz oscillator model multiplied with the Tauc Joint Density of states. The imaginary part is given as follows:

$$\mathrm{Im}(\epsilon_{\mathrm{TL}}) = \frac{A}{\omega}\frac{\omega_0\gamma(\omega-\omega_{\mathrm{g}})^2}{(\omega^2-\omega_0^2)^2+\gamma^2\omega^2}\Theta(\omega-\omega_{\mathrm{g}}) \qquad \text{(S1.1)}$$

Accordingly, ω_0 refers to the oscillator resonance frequency with the damping γ and the resonator strength A. The band gap frequency is given by ω_g. The real part can be calculated via Kramers-Kronig relation with the effective polarizability ϵ_∞.

Furthermore, due to the free charge carriers for crystalline IST, an additional Drude term must be added:

$$\epsilon_{\mathrm{Drude}} = -\frac{\omega_{\mathrm{p}}^2}{\omega^2+i\omega\gamma_{\mathrm{D}}} \qquad \text{(S1.2)}$$

The plasma frequency is denoted with ω_p, and the damping with γ_D.

The corresponding parameters of the Tauc-Lorentz-Drude model for amorphous and crystalline IST are shown in Table S1. The dielectric function is displayed in Figure 1A in the main text.

Table S1. Tauc-Lorentz-Drude model parameters for IST.

	amorphous IST	crystalline IST
A [Hz]	$13.0\cdot10^{16}$	$4.0\cdot10^{16}$
ω_0 [Hz]	$4.1\cdot10^{15}$	$4.1\cdot10^{15}$
ω_g [Hz]	$0.9\cdot10^{15}$	0
γ [Hz]	$5.2\cdot10^{15}$	$4.1\cdot10^{15}$
ω_p [Hz]	-	$7.0\cdot10^{15}$
γ_D [Hz]	-	$0.5\cdot10^{15}$
ε_∞	2	1.4

Supplementary Note 2: Polariton fitting

A sketch of the experimental situation for polariton imaging is shown in Supplementary Figure 2.1. The local electric field probed by the SNOM tip E_{tip} is given by the interference of the illuminating light E_{i} and the surface phonon polariton field E_{p}: $E_{\mathrm{tip}} = E_{\mathrm{i}} + E_{\mathrm{p}}$. Polaritons can be launched at the tip and at the interface between the crystalline and amorphous IST.

Here, two contributions to E_{p} are considered: The electric field from polaritons launched at the boundary $E_{\mathrm{p,b}}$ and the field from polaritons launched at the tip and reflected at the interface $E_{\mathrm{p,tb}}$. Combining these contributions yields $E_{\mathrm{p}} = E_{\mathrm{p,tb}} + E_{\mathrm{p,b}}$.

Tip-launched polaritons have a contribution $E_{\mathrm{p,t}} = \eta_{\mathrm{t}} E_{\mathrm{i}}$, where η_{t} describes the launching efficiency of the tip.[2,3] For the tip-launched polaritons reflected at the boundary, a propagation term has to be added: $E_{\mathrm{p,tb}} = \eta_{\mathrm{t}} E_{\mathrm{i}} r_{\mathrm{b}} \exp(i 2 k_x (x - x_0))$, where r_{b} is a reflection coefficient, k_x is the polariton wavevector, x is the position of the tip, and x_0 is the position of the boundary.[2,3]

The incident field at the boundary is given by $E_{\mathrm{i,b}} = E_{\mathrm{i}} \exp\big(-i k_{\mathrm{i}} \sin(\alpha)\,(x - x_0)\big)$, where $k_{\mathrm{i}} = k_{\mathrm{i,0}} \sqrt{\epsilon_{\mathrm{aIST}}}$, with $k_{\mathrm{i,0}} = 2\pi\nu$, is the incident wavevector in the amorphous IST, and α is the angle of incidence relative to the sample normal. The field at the tip resulting from the boundary-launched SPhP is then given by $E_{\mathrm{p,b}} = \eta_{\mathrm{b}} E_{\mathrm{i,b}} \exp(i(k_x(x - x_0) + k_z z + \varphi_{\mathrm{e}}))$, where φ_{e} is an excitation phase.[4]

The backscattered electric field from the tip at the detector is given by $E = \alpha_{\mathrm{eff}} E_{\mathrm{tip}}$. The effective polarizability strongly depends on the tip-sample distance, and the tapping amplitude is in general significantly smaller than the extent of the polariton fields in z-direction. This means that the influence of the z-dependence of the polariton fields on the detector signal is negligible compared to the z-dependence of α_{eff}, and overall, the local field

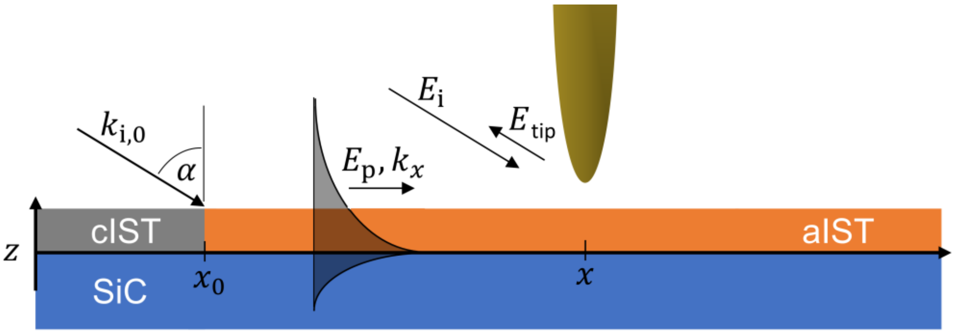

Supplementary Figure 2.1: Sketch of polariton imaging. The incident light $(k_{\mathrm{i,0}}, E_{\mathrm{i}})$ illuminates the SNOM tip at position x and the boundary of cIST/aIST at position x_0. A surface phonon polariton with wavevector k_x is launched. The electric field E_{tip} is scattered back from the tip.

at the tip can be approximated by the field at z = 0. Therefore, the nth harmonic of the total electric field at the detector is given by[4]

$$E_n = \alpha_{\mathrm{eff},n} E_{\mathrm{i}}\left(1 + \eta_{\mathrm{t}} r_{\mathrm{b}} e^{i2k_x(x-x_0)} + \eta_{\mathrm{b}} e^{i[(k_x - k_{\mathrm{i}}\sin(\alpha))(x-x_0)+\varphi_{\mathrm{e}}]}\right), \quad \text{(S2.1)}$$

with $k_{\mathrm{i}} = 2\pi\nu\sqrt{\epsilon_{\mathrm{aIST}}}$.

To estimate the polariton wavevector from a SNOM scan, the nth demodulation order optical near-field amplitude s_n is referenced to the amplitude at a position where no polaritons are present, in this case $s_n(cIST)$. The result, $s_n/s_n(cIST)$, is then given by the absolute value of the referenced electric field $|E_n/(\alpha_{\mathrm{eff},n}E_{\mathrm{i}})|$.[4]

Since the factors η_{t} and r_{b} in the term $\eta_{\mathrm{t}} r_{\mathrm{b}}$ from (S2.1) cannot be separated during fitting, they are combined into $\eta_{\mathrm{tb}} = \eta_{\mathrm{t}} r_{\mathrm{b}}$. A linear background was used to improve the fits, resulting in a function:

$$\mathrm{f} = \left|1 + \eta_{\mathrm{t}} + \eta_{\mathrm{tb}} e^{i2k_x(x-x_0)} + \eta_{\mathrm{b}} e^{i[(k_x - 2\pi\nu\sqrt{\epsilon_{\mathrm{aIST}}}\sin(\alpha))(x-x_0)+\varphi_{\mathrm{e}}]} + b(x-x_0)\right|, \quad \text{(S2.2)}$$

with the fitting parameters k_x, η_{t}, η_{tb}, η_{b}, b and φ_{e}.

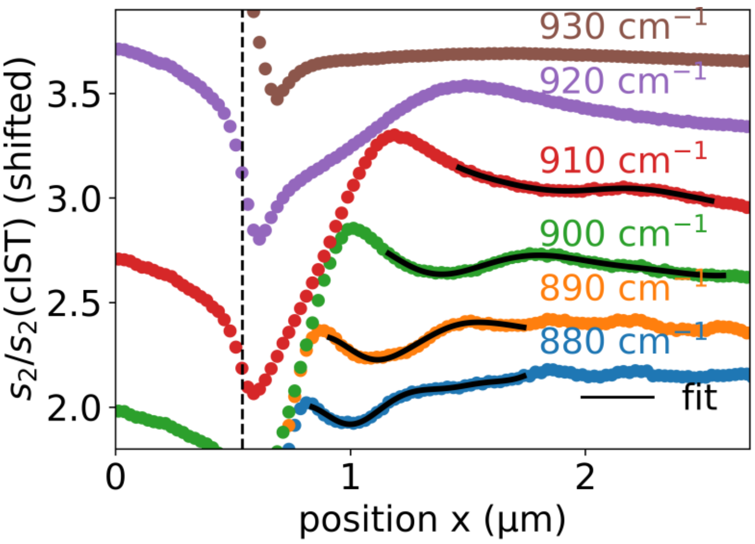

Supplementary Figure 2.2: Zoom-in to the fits presented in Figure 2C.

In the zoom-in to the fits from Supplementary Figure 2.2 more peaks appear especially for small frequencies at 880 cm^{-1} and 890 cm^{-1}. These peaks are omitted from the polariton fit since they are only topographical artifacts. The near-field amplitude line profiles and the corresponding topography line profile averaged over 10 lines are displayed together with the topography image in Supplementary Figure 2.3. Strong minima in the topography caused by

scratches at 1.8 µm, 2.2 µm and 2.6 µm exactly coincide with narrow peaks in the near-field amplitude. At the trenches with a lower topography, the SNOM tip is closer to the IST layer leading to more pronounced near-fields detected by the SNOM and therefore causing peaks in the observed near-field amplitude. Consequently, we assign those features to measurement artifacts which are not caused by polaritons.

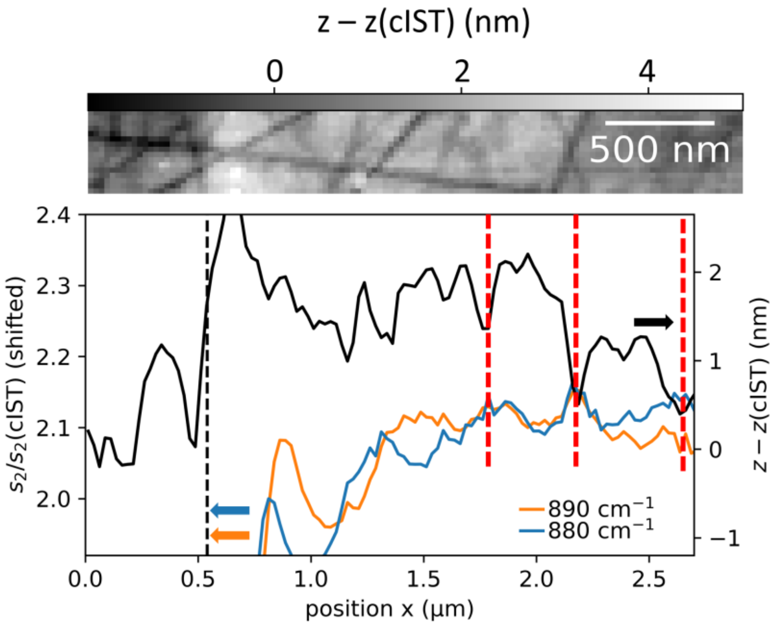

Supplementary Figure 2.3: Topography line profile (black line) in combination with near-field amplitude line profiles (colored lines). Strong minima in the topography exactly coincide with narrow peaks in the near-field amplitude (highlighted with red dashed lines).

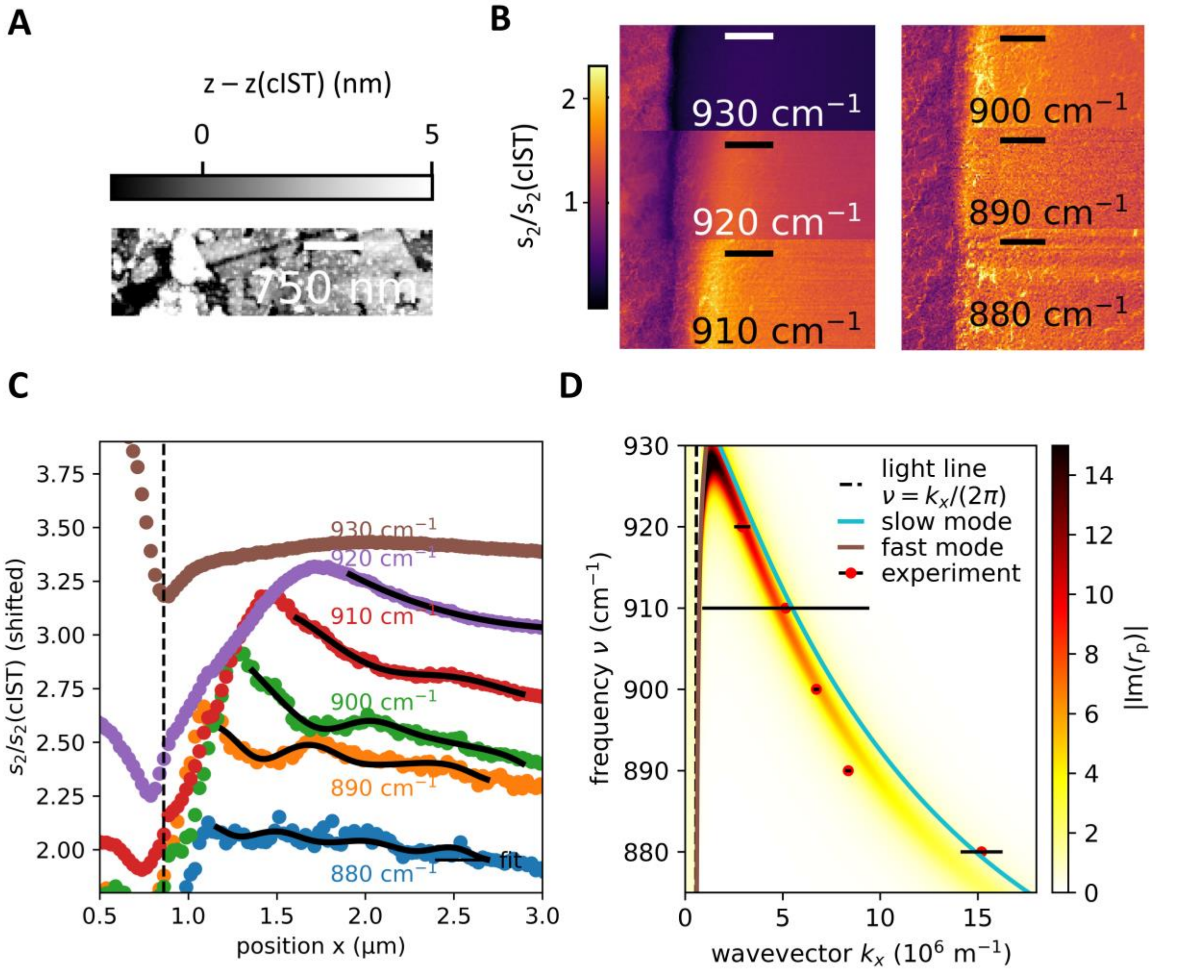

Supplementary Figure 2.4: Additional data set of propagating SPhPs. A) Topography image of another crystalline IST edge. Scratches and other particles disturb the obtained image. **B)** Measured near-field amplitude images at varied excitation frequencies. **C)** Extracted line profiles and the fit of the damped harmonic oscillation. **D)** Calculated polariton dispersion with the experimentally obtained polariton wavevectors.

Supplementary Note 3: SPhP Dispersion

The SPhP dispersion relation for the given layer stack is obtained via two ways.

First, which only works for three layers, is to derive the reflection coefficient for a (thin) homogeneous dielectric film and solve the implicit equation:

$$e^{-2k_{2,z}d} = \frac{\frac{k_{1,z}}{\epsilon_1} + \frac{k_{2,z}}{\epsilon_2}}{\frac{k_{1,z}}{\epsilon_1} - \frac{k_{2,z}}{\epsilon_2}} \cdot \frac{\frac{k_{3,z}}{\epsilon_3} + \frac{k_{2,z}}{\epsilon_2}}{\frac{k_{3,z}}{\epsilon_3} - \frac{k_{2,z}}{\epsilon_2}}, \qquad \text{(S3.1)}$$

which describes the points at which the reflection coefficient diverges.[5–7] Here, ϵ_l is the effective permittivity in layer l, d is the thickness of the second layer (the first and third layer are assumed to be infinitely thick), and $k_{l,z}^2 = k_{\mathrm{sp}}^2 - k_0^2\epsilon_l$, where k_{sp} is the SPhP wavevector and k_0 is the wavevector of the incident light in vacuum. Since the effective permittivity is a function of the frequency ν, this equation can be solved numerically, yielding a dispersion relation $\nu(k_{\mathrm{sp}})$ or $k_{\mathrm{sp}}(\nu)$.

There are three relevant solutions:[2] For the case of a localized SPhP, for example confined in a resonator, k_{sp} can be determined by the resonance condition and solving eq. S3.1 numerically for $\nu(k_{\mathrm{sp}})$ yields the resonant frequency. If propagating SPhPs are considered, the frequency of the SPhP is defined by the exciting light[2]. Solving for k_{sp} yields two results, called slow and fast SPhP, respectively[8]. The wavevectors of the fast SPhP are close to the light line $|k_{\mathrm{sp}}| \approx |k_0\sqrt{\epsilon_l}|$ while the slow SPhP can have very large wavevectors with $|k_{\mathrm{sp}}| \gg |k_0\sqrt{\epsilon_l}|$.

Second, the reflection coefficient for p-polarized light r_{p} is calculated for different wavevectors and wavenumbers with the transfer matrix method (TMM), in which the electric field in the top layer is related to the field in the bottom layer by matrices describing propagation within the different layers and transmission between them.[9,10] The dispersion relation is then given by the points at which the absolute value of the imaginary part of r_{p}, $|\mathrm{Im}(r_{\mathrm{p}})|$, diverges.[7]

In Supplementary Figure 3.1, the solutions corresponding to the slow (green line) and fast (brown line) SPhP (solved for $k_{\mathrm{sp}}(\nu)$) and the solution corresponding to a localized (blue line) SPhP (solved for $\nu(k_{\mathrm{sp}})$) for a three-layer case of SiC/aIST/air and the absolute value of $\mathrm{Im}(r_{\mathrm{p}})$ calculated with TMM for a layer stack of SiC/aIST/ZnS:SiO_2/air are plotted. The thicknesses of the aIST and the ZnS:SiO_2 layers were set to 35 nm and 15 nm, respectively. The higher

effective permittivity of the capping layer compared to air shifts the dispersion to lower frequencies. This effect becomes even more pronounced if the thickness of the capping layer is increased (see the calculated dispersion curves for varied capping layer thickness in Supplementary Figure 3.2). Another possibility to increase the polariton confinement is reducing the IST layer thickness (see Supplementary Figure 3.3), revealing values up to 125 for

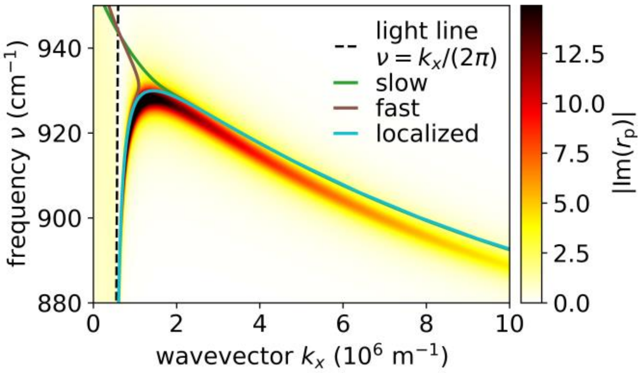

Supplementary Figure 3.1: The three solutions of the dispersion relation for a three-layer case (SiC/aIST/air) and the dispersion calculated with TMM for the layer stack SiC/aIST/ZnS:SiO_2/air. The solution of localized SPhPs equals the fast (slow) SPhP for small

a 7 nm thin IST layer.

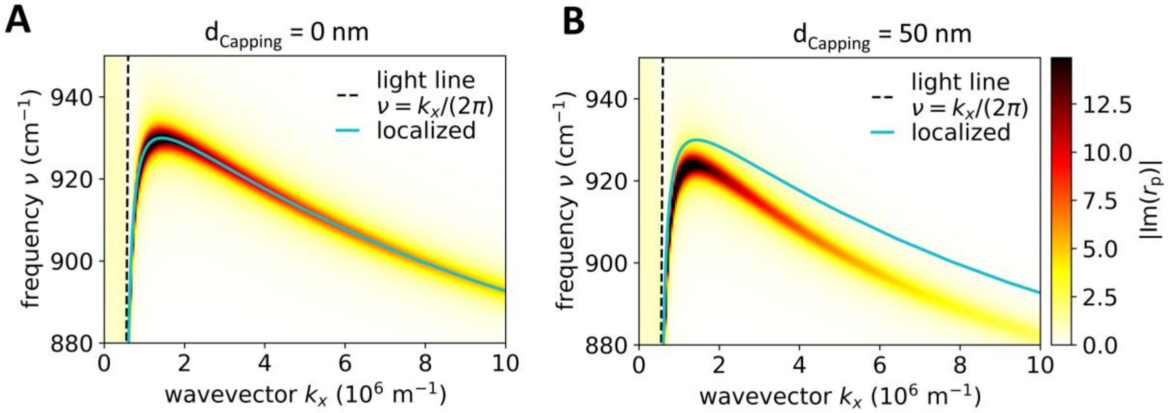

Supplementary Figure 3.2: Calculated SPhP dispersion for varied capping thicknesses of 0 (A) and 50 nm (B)

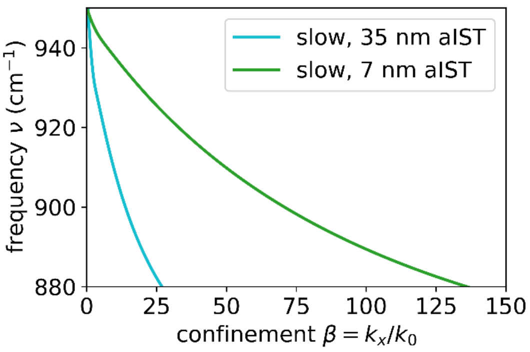

Supplementary Figure 3.3: Calculated polariton confinement for different IST layer thicknesses.

Supplementary Note 4: Writing procedure of confined resonators

Confined SPhP resonators are directly optically written inside the amorphous IST layer with precisely arranged laser pulses. The schematic working principle is shown in Supplementary Figure 4. First, multiple crystallization spots are positioned next to each other with vertical distance Δy = 1 µm and horizontal distance Δx = 1.3 µm (i). The larger horizontal distance is caused by the intrinsic elliptical shape of the laser beam. Afterwards, 25 spots circularly arranged are added, resulting in the shown circular cavities from Figure 3 (ii). Finally, these cavities are locally addressed again, and the diameter is reduced by applying again 25 spots within a smaller circle (iii). All spots overlap and hence result in a homogeneous crystalline area with cavities of amorphous IST in the center.

For programming these resonators, the size is not limited by the spot size, but rather by the step size (accuracy/resolution) of the applied piezo actuators used for positioning the sample and consequently in the nanometer range. It is even possible by combining sophisticated spatially overlapping crystallization and reamorphization pulses to tailor the size of the resonators at will down to a few hundred nanometers.[11]

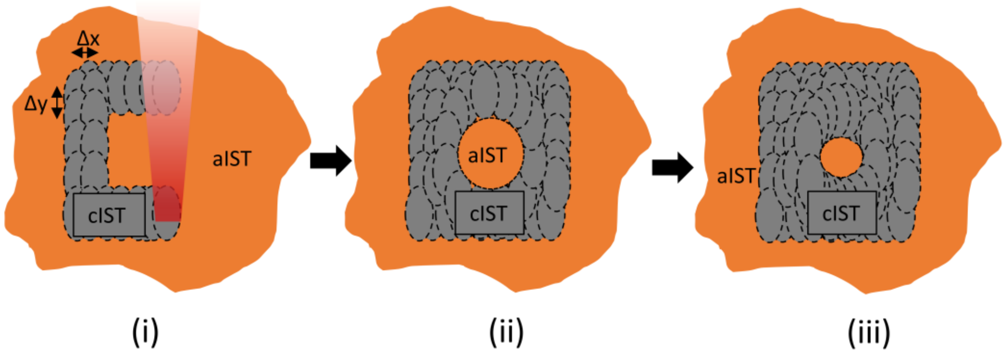

Supplementary Figure 4: Schematic writing process of confined resonators.

We chose our applied method to fabricate the resonator structures instead of starting from a thermally crystallized IST layer and applying reamorphization pulses because spatially tailoring crystallization with laser spots offers a larger degree of freedom to test various crystallization parameters. Moreover, the fixed amount of SiC substrates limited our possibilities to investigate a test series of different thermal crystallization parameters for homogeneous layers.

Supplementary Note 5: Images of cavities with different diameters

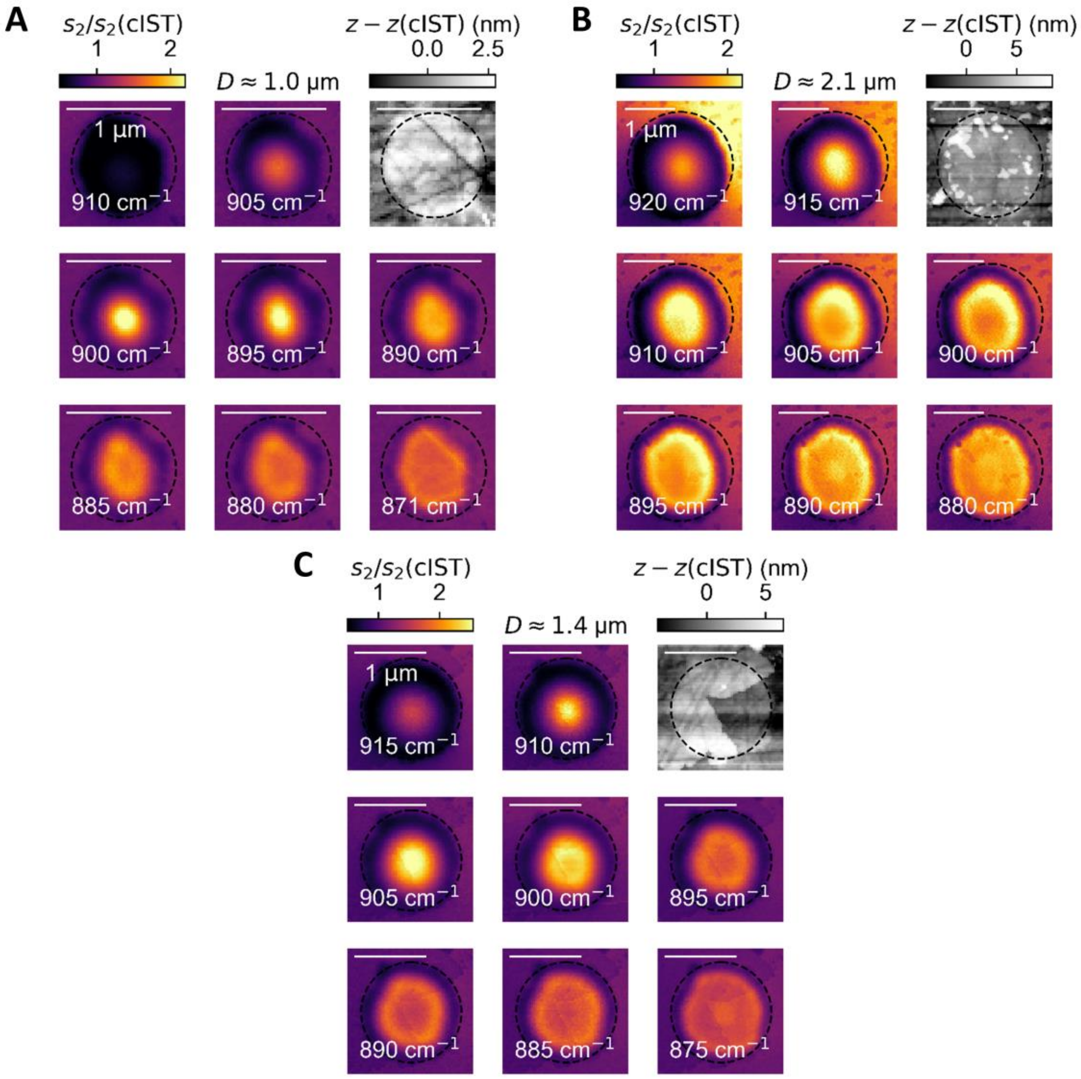

Supplementary Figure 5.1: SNOM images and topography of cavities with diameters of about 1 µm, 2.1 µm and 1.4 µm. The circles indicate the cavity boundary. Due to dirt and other surface contaminations of the $ZnS:SiO_2$, the visibility of the cavities in the AFM images is low.

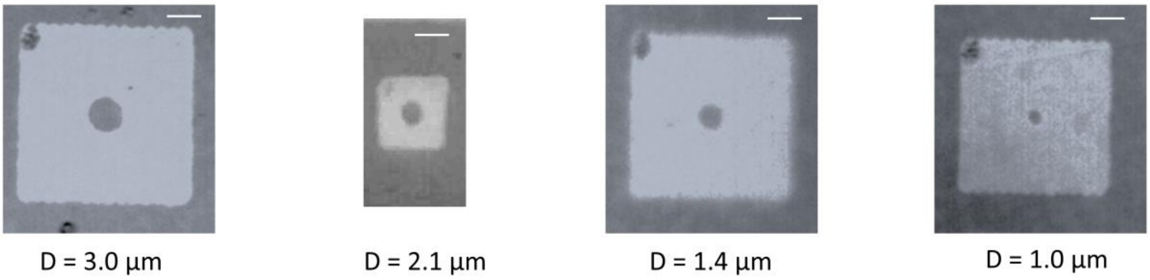

Supplementary Figure 5.2: Light microscope images of the different cavities. The crystallized IST appears bright while the amorphous IST is significantly darker. The scale bars equal 3 µm.

Supplementary Note 6: Field simulations of the cavities with varied diameters

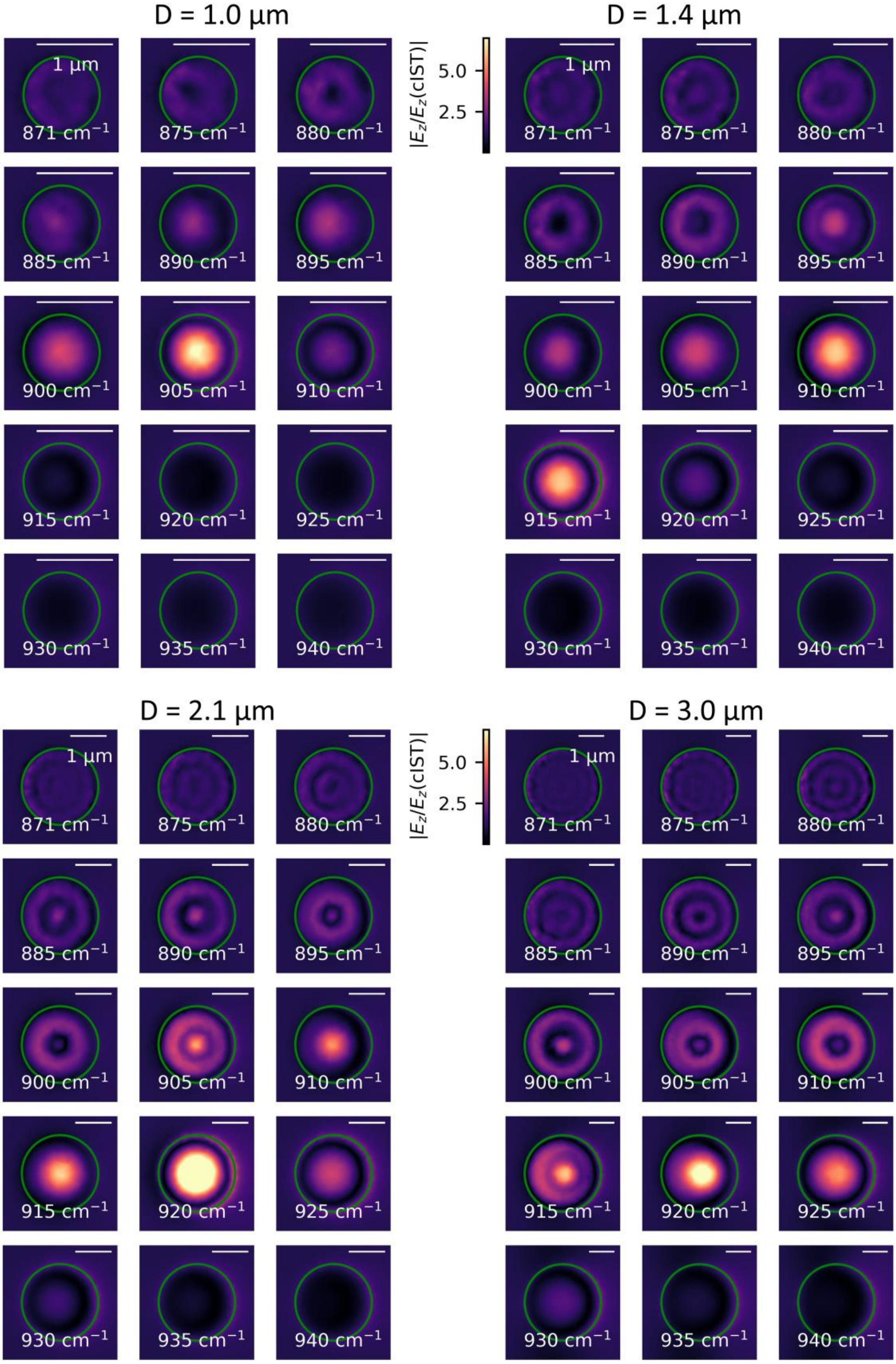

Supplementary Figure 6: Numerical field simulations of the out-of-plane electric field for varied cavity diameters and excitation frequencies.

Supplementary Note 7: Quality factor of the resonators

The quality factor of the resonators is determined by fitting a Lorentzian function to the optical near-field amplitude contrasts at varied excitation frequencies for the different cavity diameters (see solid curves in Supplementary Figure 7). Due to the strong asymmetry of the measured data points, only two points left to the maximum are taken into account. The determined quality factors are in the range of 50, except for the orange curve which shows a quality factor of 31.

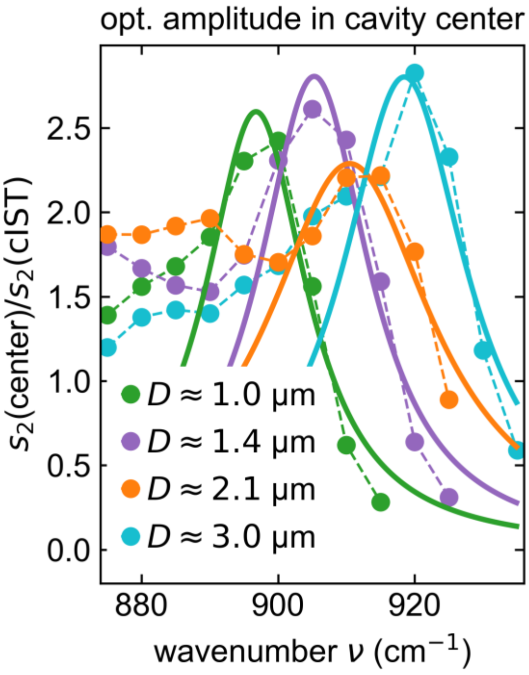

Supplementary Figure 7: Fitted Lorentzian functions (solid curves) to the experimentally obtained near-field amplitude contrasts. The obtained quality factors for D = 1.0 µm, 1.4 µm, 2.1 µm, and 3.0 µm are 50, 46, 31, and 42, respectively.

Supplementary Note 8: Analytical model of circular resonators

For a circular resonator or cavity with diameter D, the resonance condition for a mode (m, n) is given by

$$k_{\mathrm{sp}}(\nu)D + \phi = 2x_n(J_m), \tag{S7.1}$$

where ϕ is the phase increment caused by the reflection at the cavity boundary and $x_n(J_m)$ is the nth zero of the Bessel function of the first kind J_m.[12]

For a given cavity diameter, $k_{\mathrm{sp}}(\nu)$ can be calculated for different modes with eq. S7.1. Afterwards, the resonant frequency is determined by the point where $|\mathrm{Im}(r_{\mathrm{p}})|$, calculated with the TMM for four layers (air/capping/IST/SiC), is maximal for the given $k_{\mathrm{sp}}(\nu)$.

The resonant frequency in dependence of the cavity diameter for the (0, 1) mode and two reflection phases is shown in Supplementary Figure 8, together with the experimental data. The experimental resonance positions were estimated from the SNOM images (Figure 3 and Supplementary Figure 6) and fit to the analytical model if a reflection phase $\phi = -\pi$ is assumed.

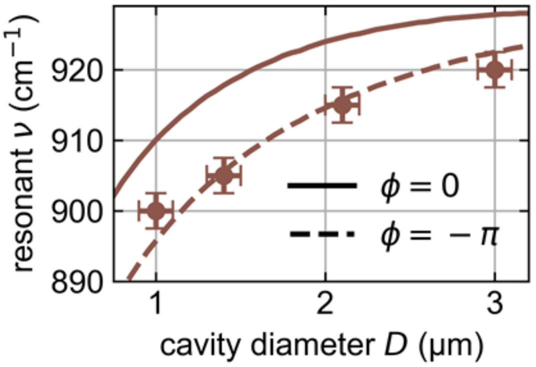

Supplementary Figure 8: Analytical relationship between cavity diameter and resonant frequency for different reflection phases and experimental data.

Supplementary Note 9: Resonator arrays investigated in the far-field

For three selected diameters, multiple cavities with the same diameter were arranged in an array with an area of about 40 x 40 µm^2. Optical images of these arrays are shown in Supplementary Figure 9A. The arrays were measured with a Fourier-transform infrared microscope in a grazing incidence reflection setup, with incidence angles ranging from 52° to 84°.[13]

The amorphous surrounding was blocked out with knife-edge apertures. Supplementary Figure 9B displays the measured spectra, which are shifted for better visibility and were referenced to an area of crystallized IST. In each spectrum, a dip in reflectance (marked by colored arrows) is visible, caused by the SPhP resonance in the cavity. The near-field resonance position does not coincide with the far-field one. This behavior is known from theory and experiments.[14]

The spectra in Supplementary Figure 9C were simulated with CST, under the assumption of an incidence angle of 70°. Sharp dips in the reflectance curves corresponding to the SPhP resonances coincide with the experimentally obtained curves. In general, a good agreement between simulated and the experimentally observed spectra is present. The lower magnitude and increased broadness of the experimental peaks can be attributed to the broad angular

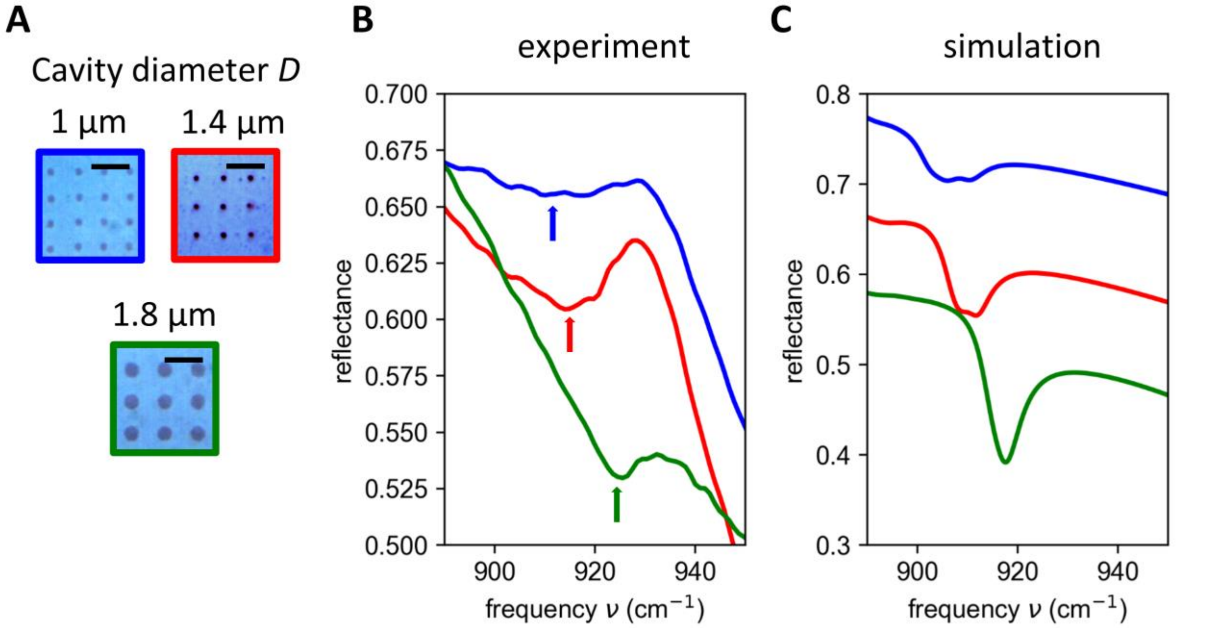

Supplementary Figure 9. Far-field measurements and simulations of cavity arrays. **A)** Optical microscope images of a section of the arrays of resonators with different diameters. The scale bars correspond to 5 µm. **B), C)** Experimental **(B)** and simulated **(C)** reflectance. The arrows are a guide to the eye and indicate dips in the reflectance corresponding to the cavity resonance.

distribution of the objective used and imperfect varying resonators across the array, especially a varying diameter and no perfectly round borders due to the stochastic nature of the crystallization. To improve the agreement between experiment and simulations, we modified the simulated unit cell of the SPhP resonators. In particular, we simulated three resonators within the same unit cell with slightly different diameters to take fabrication imperfections into account which led to broader resonances as visible in the measured reflectance spectra.

Supplementary Note 10: Comparative Study with previous literature

Finally, we performed a comparative study with previous related work in literature to evaluate the different strengths of our method. We choose several characteristics such as reconfigurability, field confinement and quality factor. The results are displayed in Table S2.

Table S2. Comparative study of polariton resonators

Publication	material	Near-/<br>Farfield	Prop.<br>length	Field confinement<br>Quality factor	Mode<br>analysis	Reconfi-<br>gurability
This work	SiC	Both	~ 1 µm	λ/35<br>Q ~ 50	yes	Modify shapes
Li et al.[8]	SiO_2	Both	~ 1 µm	λ/30 for d=30 nm<br>λ/80 for d=7 nm<br>Q ~ 95	yes	Erase and write
Sumikura et al.[2]	SiC	Nearfield	~ 1 µm	λ/50	yes	Turn on and off
Wang et al.[15]	SiC	Both	-	Q ~ 50-70	yes	No
Tamagnone et al.[16]	hBN<br>α-MoO_3	Nearfield<br>Both	-<br>-	Q ~ 300<br>Q ~ 250	yes<br>no	No<br>No
Duan et al.[17]	hBN	Nearfield	-	λ/42<br>Q ~ 165	yes	graphene<br>doping
Folland et al.[18]	hBN	Nearfield	~ 1-3 µm	-	-	Heating VO_2
Sheinfux et al.[19]	hBN	Nearfield	-	Q ~ 50-480	yes	No

Overall, our optically written resonators show similar performance compared to previous work in literature based on bulk polar crystals like SiC with the unprecedented advantage of easy reconfigurability to modify the resonator shapes to tune the field confinement. We anticipate even better performance by combining our concept with thinner IST films or low-loss 2d materials such as hBN or α-MoO_3.[20]

Supplementary Note 11: Reamorphization of cavities

Previously, the cavities have been programmed by crystallizing the amorphous IST around the cavity (see Supplementary Figure 11A). To demonstrate the reversibility of the optical writing, cavities were created by crystallizing the whole area and then reamorphizing a circular area in the center. This process is sketched in Supplementary Figure 11B***.*** In Supplementary Figure 11D, SNOM images of such a cavity are compared to the images of cavity with a diameter $D \approx 1.4$ µm (Supplementary Figure 11C), fabricated with the initial method (c.f. Supplementary Figure 11C). The SNOM images look similar, but deformation of the sample (see Supplementary Figure 11E) and resulting higher average distance to the SiC/IST interface, where the polariton field is highest, leads to reduced signal and visibility. A possible explanation for the deformation is that the IST expands rapidly when heated, thus deforming and possibly cracking the capping.[21] At 880 cm^{-1}, the ring-like feature with higher signal cannot be discerned anymore. An optimization of the laser parameters might lead to decreased deformation and therefore increased reversibility.

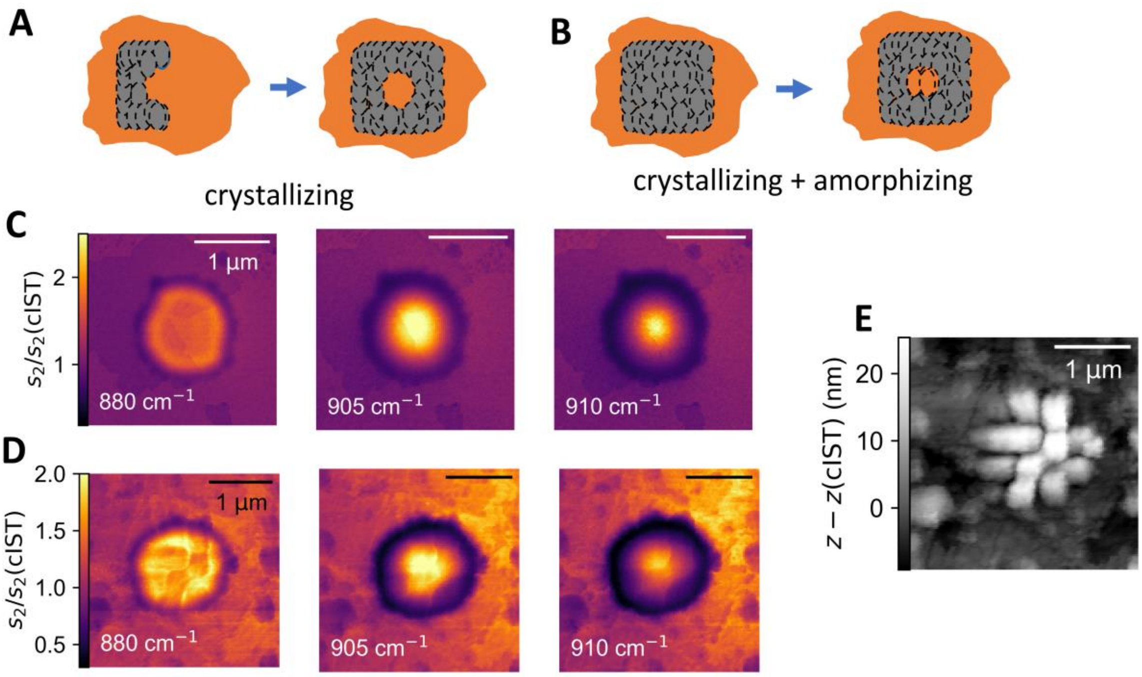

Supplementary Figure 11. Reamorphized cavities. **A), B)** Sketch of the optical writing process used to define the cavities with the help of crystallizing (**A**) or crystallizing and amorphizing (**B**) pulses. The aIST is colored red, cIST blue. **C), D)** SNOM images at different frequencies of the cavities created without (**C**) and with (**D**) amorphizing pulses. **E)** Topography of the reamorphized cavity.

Supplementary Note 12: Hyperbolic Phonon Polariton cavities with hBN

We demonstrated our concept of direct programming SPhP resonators inside IST on the polar crystal SiC. However, the concept can be easily transferred to other material systems such as 2d hyperbolic materials, e.g., hexagonal boron nitride (hBN). Therefore, we performed numerical simulations of an amorphous IST cavity with a diameter D = 750 nm buried below a 100 nm thin hBN layer (see Supplementary Figure 12A). The dielectric function of hBN with the lower and upper Reststrahlenband is shown in Supplementary Figure 12B. The upper Reststrahlenband is located between 1360-1610 cm^{-1} with $Re(\varepsilon_z) > 0$ and $Re(\varepsilon_t) < 0$ showing a hyperbolic response. Normalized electric field simulations of the E_z-component for three different excitation frequencies are displayed in Supplementary Figure 12C. Inside the cavities similar mode patterns compared to the IST resonators on SiC can be observed. Outside of the resonators, concentric rings corresponding to propagating polaritons are visible.

Hence, the concept of programming confined resonators inside the PCM is not limited to the polar crystal SiC but can be easily transferred to other materials such as hBN or anisotropic crystals.[22–24]

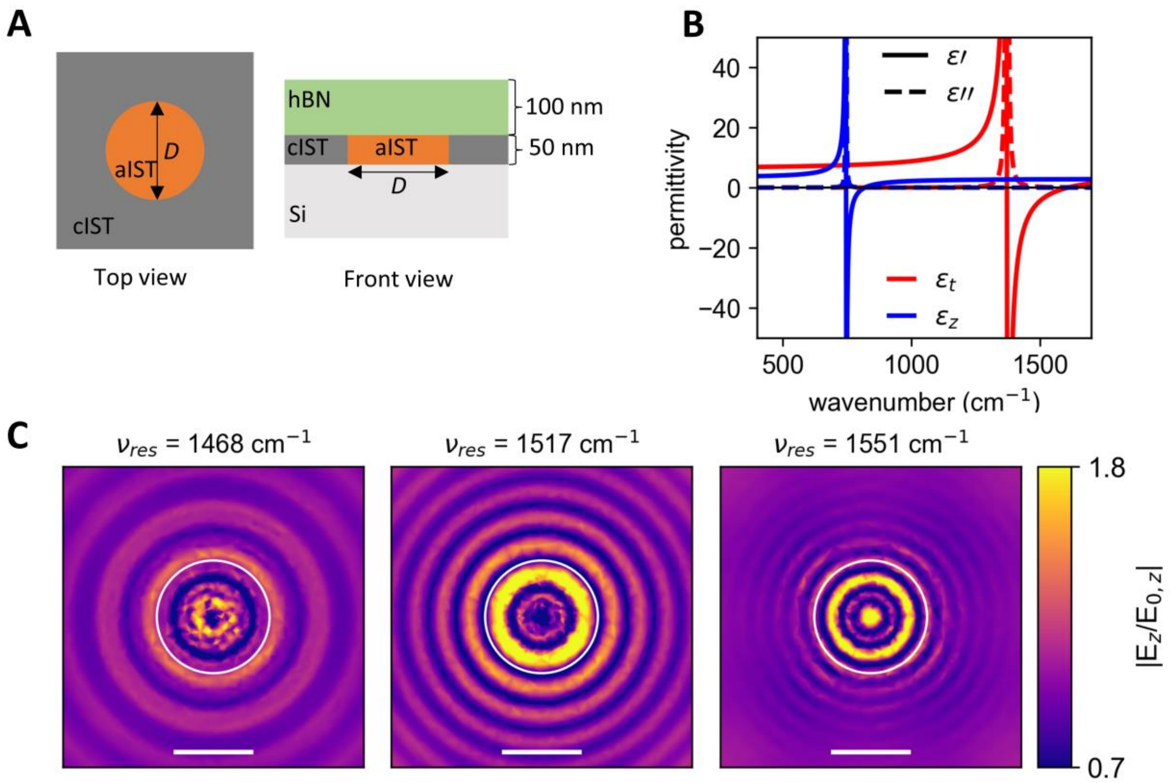

Supplementary Figure 12: Simulations of IST cavities below an hBN flake. A) Schematic sketch of the simulated layerstack with an amorphous circular cavity with a diameter D = 750 nm buried below the 100 nm hBN. **B)** Dielectric function of the in-plane ε_t and out-of-plane ε_z

component. **C)** Simulated out-of plane component of the electric field normalized to the incident field 1 nm above the hBN for frequencies in the upper Reststrahlenband. Polariton fringes are clearly visible, as well as different modes inside the cavity. The scale bars equal 500 nm.

References

(1) Heßler, A. et al. In3SbTe2 as a programmable nanophotonics material platform for the infrared. *Nature Communications* **2021**, *12* (1), 924. DOI: 10.1038/s41467-021-21175-7.

(2) Sumikura, H. et al. Highly Confined and Switchable Mid-Infrared Surface Phonon Polariton Resonances of Planar Circular Cavities with a Phase Change Material. *Nano Letters* **2019**, *19* (4), 2549–2554. DOI: 10.1021/acs.nanolett.9b00304.

(3) Dai, S. et al. Tunable Phonon Polaritons in Atomically Thin van der Waals Crystals of Boron Nitride. *Science* **2014**, *343* (6175), 1125–1129. DOI: 10.1126/science.1246833.

(4) Huber, A.; Ocelic, N.; Kazantsev, D.; Hillenbrand, R. Near-field imaging of mid-infrared surface phonon polariton propagation. *Appl. Phys. Lett.* **2005**, *87* (8), 81103. DOI: 10.1063/1.2032595.

(5) Born, M.; Wolf, E. *Principles of Optics: Electromagnetic Theory of Propagation, Interference and Diffraction of Light*, 7th ed.; Cambridge University Press, 1999. DOI: 10.1017/CBO9781139644181.

(6) Maier, S. *Plasmonics: Fundamentals and Applications*; Springer US, 2007.

(7) Raether, H. *Surface Plasmons on Smooth and Rough Surfaces and on Gratings*, 1st ed.; Springer Berlin, Heidelberg, 1988. DOI: 10.1007/BFb0048317.

(8) Li, P. et al. Reversible optical switching of highly confined phonon–polaritons with an ultrathin phase-change material. *Nature Materials* **2016**, *15* (8), 870–875. DOI: 10.1038/nmat4649.

(9) Zhan, T.; Shi, X.; Dai, Y.; Liu, X.; Zi, J. Transfer matrix method for optics in graphene layers. *Journal of Physics: Condensed Matter* **2013**, *25* (21), 215301. DOI: 10.1088/0953-8984/25/21/215301.

(10) Zi, J.; Wan, J.; Zhang, C. Large frequency range of negligible transmission in one-dimensional photonic quantum well structures. *Appl. Phys. Lett.* **1998**, *73* (15), 2084–2086. DOI: 10.1063/1.122385.

(11) Heßler, A.; Conrads, L.; Wirth, K. G.; Wuttig, M.; Taubner, T. Reconfiguring Magnetic Infrared Resonances with the Plasmonic Phase-Change Material In3SbTe2. *ACS Photonics* **2022**, *9* (5), 1821–1828. DOI: 10.1021/acsphotonics.2c00432.

(12) Filter, R.; Qi, J.; Rockstuhl, C.; Lederer, F. Circular optical nanoantennas: an analytical theory. *Phys. Rev. B* **2012**, *85* (12), 125429. DOI: 10.1103/PhysRevB.85.125429.

(13) Maß, T. W. W. Concepts for improving surface-enhanced infrared spectroscopy. Dissertation, RWTH Aachen University, 2018.

(14) Alonso-González, P. et al. Experimental Verification of the Spectral Shift between Near- and Far-Field Peak Intensities of Plasmonic Infrared Nanoantennas. *Phys. Rev. Lett.* **2013**, *110* (20), 203902. DOI: 10.1103/PhysRevLett.110.203902.

(15) Wang, T.; Li, P.; Hauer, B.; Chigrin, D. N.; Taubner, T. Optical Properties of Single Infrared Resonant Circular Microcavities for Surface Phonon Polaritons. *Nano Letters* **2013**, *13* (11), 5051–5055. DOI: 10.1021/nl4020342.

(16) Tamagnone, M. et al. High quality factor polariton resonators using van der Waals materials. *arXiv [physics.optics]* **2020**, *1905.02177v2*. DOI: 08611.

(17) Duan, J. et al. Active and Passive Tuning of Ultranarrow Resonances in Polaritonic Nanoantennas. *Adv. Mater.* **2022**, *34* (10), 2104954. DOI: 10.1002/adma.202104954.

(18) Folland, T. G. et al. Reconfigurable infrared hyperbolic metasurfaces using phase change materials. *Nature Communications* **2018**, *9* (1), 4371. DOI: 10.1038/s41467-018-06858-y.

(19) Herzig Sheinfux, H. et al. High-quality nanocavities through multimodal confinement of hyperbolic polaritons in hexagonal boron nitride. *Nature Materials* **2024.** DOI: 10.1038/s41563-023-01785-w.

(20) Lu, D. et al. Tunable hyperbolic polaritons with plasmonic phase-change material In3SbTe2. *Nanophotonics* **2024.** DOI: 10.1515/nanoph-2023-0911.
(21) Heßler, A. Optical programming of infrared phase-change material metasurfaces. Dissertation, RWTH Aachen University, 2022.
(22) Ma, W. et al. In-plane anisotropic and ultra-low-loss polaritons in a natural van der Waals crystal. *Nature* **2018**, *562* (7728), 557–562. DOI: 10.1038/s41586-018-0618-9.
(23) Zheng, Z. et al. A mid-infrared biaxial hyperbolic van der Waals crystal. *Science Advances* **2019**, *5* (5), eaav8690. DOI: 10.1126/sciadv.aav8690.
(24) Passler, N. C. et al. Hyperbolic shear polaritons in low-symmetry crystals. *Nature* **2022**, *602* (7898), 595–600. DOI: 10.1038/s41586-021-04328-y.
